# Reduced Activity of the Aortic Gamma-Glutamyltransferase Does Not Decrease *S*-Nitrosoglutathione Induced Vasorelaxation of Rat Aortic Rings

**DOI:** 10.3389/fphys.2016.00630

**Published:** 2016-12-20

**Authors:** Caroline Perrin-Sarrado, Marios Pongas, Fatima Dahboul, Pierre Leroy, Alfonso Pompella, Isabelle Lartaud

**Affiliations:** ^1^EA3452 CITHEFOR “Drug Targets, Formulation and Preclinical Assessment”, Faculté de Pharmacie, Université de LorraineNancy, France; ^2^Department of Translational Research and of New Surgical and Medical Technologies, University of Pisa Medical SchoolPisa, Italy

**Keywords:** NO-dependent vasorelaxation, Spontaneous Hypertensive Rat, *S*-nitrosoglutathione, gamma-glutamyltransferase, aortic ring

## Abstract

**Aims:** Gamma-glutamyl transferase (GGT), an enzyme present on the endothelium, is involved in the release of nitric oxide (NO) from *S*-nitrosoglutathione (GSNO) and in the GSNO-induced vasodilation. Endogenous GSNO is a physiological storage form of NO in tissues while exogenous GSNO is an interesting candidate for compensating for the decreased NO bioavailability occurring during cardiovascular diseases. We investigated in a rat model of human hypertension, the spontaneous hypertensive rat (SHR), submitted or not to high salt diet, whether a decreased vascular GGT activity modifies the vasorelaxant effect of GSNO.

**Methods:** Thoracic aortic rings isolated from male SHR and Wistar Kyoto rats (WKY) aged 20–22 weeks—submitted or not for 8 weeks to a high salt diet (1% w/v NaCl in drinking water) were pre-constricted with phenylephrine then submitted to concentration-vasorelaxant response curves (maximal response: E_max_; pD_2_) to carbachol or sodium nitroprusside to evaluate endothelial dependent or independent NO-induced vasodilation, or GSNO (exogenous NO vasodilation depending from the endothelial GGT activity). GGT activity was measured using a chromogenic substrate in aortic homogenates. Its role in GSNO-induced relaxation was assessed following inhibition of the enzyme activity (serine-borate complex). That of protein disulfide isomerase (PDI), another redox sensitive enzyme involved in GSNO metabolism, was assessed following inhibition with bacitracin.

**Results:** Aortic GGT activity (18–23 μmol/min/mg of tissue in adult WKY) decreased by 33% in SHR and 45% in SHR with high salt diet. E_max_ and pD_2_ for sodium nitroprusside were similar in all groups. E_max_ for carbachol decreased by −14%, reflecting slight endothelial NO-dependent dysfunction. The GSNO curve was slightly shifted to the left in SHR and in SHR with high salt diet, showing a small enhanced sensitivity to GSNO. Involvements of GGT, as that of PDI, in the GSNO effects were similar in all groups (pD_2_ for GSNO −0.5 to −1.5 following enzymatic inhibition).

**Conclusion:** Hypertension is associated with a decreased aortic GGT activity without decreasing the vasorelaxant effects of GSNO, whose bioactivity may be supplemented through the alternative enzymatic activity of PDI.

## Introduction

Many cardiovascular diseases are associated with a decreased endothelial-dependent bioavailability of nitric oxide (NO), leading to impaired vasodilation, pro-inflammatory/oxidative, pro-proliferative, and pro-thrombotic status (Vanhoutte and Boulanger, 1995; Le Brocq et al., 2008). Atherosclerosis, for example, which mainly concerns large conductance arteries where vasoactive functions depend specifically on the bioavailability of NO (Luksha et al., 2009) leads to the use of several NO donors (organic nitrates) for therapeutics. However, these treatments are known to provide fast NO release concomitant with induction of oxidative stress and tolerance (Bauer and Fung, 1991; Parker and Gori, 2001). New NO-donors, such as *S*-nitrosothiols have been proposed as interesting therapeutic alternatives as they do not present the drawbacks of organic nitrates.

*S*-nitrosoglutathione (GSNO), the nitrosated form of glutathione, is currently investigated as NO- donor to restore NO homeostasis, and is an interesting candidate for therapeutics as it mimics endogenous GSNO-related functions. Endogenous cellular formation of GSNO involves either direct reaction of NO/nitrosating species with GSH, or previous formation of protein-based or low molecular weight *S*-nitrosothiols followed by subsequent transnitrosation to GSH (Al-Sa'doni and Ferro, 2004; Broniowska et al., 2013). The ability of GSNO to transfer its NO to cystein residue of endogenous peptides or proteins *via* dynamic processes of nitrosation/denitrosation (Gaucher et al., 2013) explains its ability to store and transport NO to sites of utilization in the body (Wu et al., 2016).

Gamma-glutamyl transferase (GGT) is one of the enzyme activity implicated in the release of NO from GSNO and its uptake into the cell (Hogg et al., 1997; Bramanti et al., 2009). GGT specifically catalyzes endogenous as exogenous GSNO breakdown producing cysteinylglycine and NO in endothelial cells. There, either NO diffuses to the smooth muscle cells to activate the soluble guanylyl cyclase/cyclic guanosine monophosphate pathway and induce vasorelaxation (Tullett et al., 2001; Alencar et al., 2003; Heikal et al., 2011), or it reacts with endothelial glutathione or proteins cysteine residue to form *S*-nitrosothiols. Those *S*-nitrosothiols may release NO through transnitrosation processes involving enzymes such as the system thioredoxin/thioredoxin reductase (Marozkina and Gaston, 2012; Gaucher et al., 2013). We have previously documented that endothelial GGT is critical for GSNO-dependent NO-delivery and vasorelaxation in aortic rings isolated from normotensive rats (Dahboul et al., 2012). In this context, GSNO and its cellular metabolism enzymes are likely to be involved in blood pressure regulation (Ishibashi et al., 2011).

In the present study, we evaluated GSNO-induced vasorelaxation in the Spontaneous Hypertensive Rat (SHR), with the hypothesis that hypertension may impair GGT activity of the vessel wall. Therefore, the bioactivity of exogenous treatment with GSNO would be modified. In the present study, we analyzed concentration-vasorelaxant response curves (maximal response E_max_ and pD_2_ calculated as –log EC_50_, the half maximal effective concentration) to carbachol and sodium nitroprusside in order to evaluate endothelial dependent and independent NO-induced vasodilation, and to GSNO (exogenous NO vasodilation depending from the endothelial GGT activity). As protein disulfide isomerase (PDI), a membrane enzyme from the redoxins family, has also been reported to be involved in the release of NO from GSNO (Heikal et al., 2011), we also evaluated whether the vasorelaxant effect of GSNO may be warranted through such alternative enzymatic activity.

## Materials and methods

### Chemicals

All reagents were of analytical grade. Carbachol, phenylephrine, *L*-γ-glutamyl-3-carboxy-4-nitroanilide, and all other reagents were obtained from Sigma-Aldrich (Saint Quentin Fallavier, France). Ultrapure deionized water (18.2 MΩ.cm) was used to prepare all solutions. Standard solutions of GSNO were prepared by nitrosation of glutathione after mixing glutathione with sodium nitrite (ration 1:1) in acidic medium according to the method previously described (Parent et al., 2013).

The purity of GSNO was assessed by ultraviolet spectrophotometry using its molar absorbance at 334 nm (ε = 922 M^−1^.cm^−1^).

### Rats and ethical statements

All experiments were performed in accordance with the European Parliament guidelines (2010/63/EU) for the use of experimental animals and the respect of the 3 Rs' requirements for Animal Welfare. The protocols and procedures were approved by the advisory regional ethical committee on animal experiments: Comité d'Ethique Lorrain en Matière d'Expérimentation Animale, CELMEA protocol agreement N° 02420.03.

Young adult normotensive Wistar-Kyoto rats (WKY) or SHR (11 weeks-old, 300–325 g) were purchased from Janvier Laboratories (Le Genest St Isle, France), kept under standard conditions (temperature: 21 ± 1°C, hygrometry 60 ± 10%, light on 6 a.m. to 6 p.m.) and ate standard diet (A04, Safe, Villemoisson-sur-Orge, France) and drank water (reverse osmosis system, Culligan, Brussels, Belgium) *ad libitum*. After 1 week, they were randomly separated into two series: 20–22 weeks-old adult SHR and WKY (WKY/SHR), 20–22 weeks-old adult SHR and WKY rats submitted to a high salt diet (WKY-S/SHR-S) to impair endothelial-dependent vasodilation (Kagota et al., 2001). In high salt diet groups, salt was incorporated at 1% (w/v) in drinking water for 8 weeks from the age of 12–14 weeks.

Mean body weight was 417 ± 9 and 418 ± 9 g in WKY/SHR, 399 ± 9 and 394 ± 9 g in WKY-S/SHR-S. The mean systolic blood pressure was measured by the tail-cuff method (149 ± 5 and 225 ± 10 mmHg in WKY/SHR; 161 ± 9 and 256 ± 9 mmHg in WKY-S/SHR-S).

Rats were anesthetized with sodium pentobarbitone (60 mg.kg^−1^, intraperitoneal injection, Sanofi Santé Nutrition Animale, Libourne, France) and the adequacy of anesthesia was checked by testing the loss of the corneal and pinch paw withdrawal reflexes. If a change in the reflexes occurred, a bolus of sodium pentobarbitone was immediately administered. After administration of heparin (1000 IU.kg^−1^ heparine Choay, penis vein), rats were sacrificed by exsanguination and segments (3 cm) of the descending thoracic aorta were removed. Vessels were cleaned from surrounding connective tissues, cut into 2-mm long rings (8 rings per rat) and immediately used for vasoactivity. Some samples of aortic rings were frozen in liquid nitrogen and kept at −80°C until biochemical studies were analyzed.

### Vasorelaxation studies

Vasorelaxation was evaluated on endothelium-intact aortic rings (Dahboul et al., 2012). Aortic vasoactivity was measured using an isometric tension recording system in 10 mL organ chambers (EMKABATH, Emka Technology, France). All manipulations and assays involving GSNO were performed under conditions of subdued light, in order to minimize light-induced degradation. The bath was filled with Krebs' solution containing 119 mM NaCl, 4.7 mM KCl, 1.2 mM KH_2_PO_4_, 1.2 mM MgSO_4_, 1.6 mM CaCl_2_, 24 mM NaHCO_3_, 5.5 mM glucose, adjusted to pH 7.4 (10 mL, 37°C) and continuously bubbled with 95% O2 and 5% CO2. Following 60-min equilibration with a basal resting tension determined at 2 g, rings were exposed two times to KCl (60 mM, 5 min). Aortic rings (*n* = 7–19 per group, from 4 to 11 different rats in each group) were then preconstricted with 10^−6^ M phenylephrine. At the plateau of contraction, concentration-relaxation response curves to increasing concentrations of GSNO (10^−10^ to 3.10^−5^ M) were performed.

The roles of GGT and PDI were assessed by inhibiting their activity with competitive reversible inhibitors, the serine-borate complex (20 mM) or bacitracin (200 μM), respectively (Dahboul et al., 2012).

Endothelial dependent and independent NO-induced vasodilation were evaluated by measuring the ability for preconstricted aortic rings to relax following, respectively, administration of carbachol, a muscarinic acetylcholine receptors agonist, and sodium nitroprusside, an endothelial independent NO-release drug. Decreases in maximal response (Emax) to carbachol (10^−10^ to 10^−5^ M response curves) witness endothelial NO-related dilating dysfunction, while changes in sodium nitroprusside concentration response curves reflects dysfunction in smooth muscle cell contractile machinery (Kreye et al., 1975; Boulanger et al., 1994).

### GGT activity in aorta

GGT activity was measured spectrophotometrically after hydrolysis of the synthetic GGT substrate *L*-γ-glutamyl-3-carboxy-4-nitroanilide as previously described (PetitClerc et al., 1980). Briefly, aortic rings were homogenized and incubated for 2 h at 37°C in Tris buffer (100 mM, pH 7.4) containing 1 mM *L*-γ-glutamyl-3-carboxy-4-nitroanilide, 20 mM glycylglycine, and 10 mM MgCl2. After centrifugation at 42,000 × g for 10 min at 4°C, supernatant absorbance was read at 405 nm to monitor the release of 5-amino-2-nitrobenzoate (ε = 9500 M^−1^.cm^−1^) from *L*-γ-glutamyl-3-carboxy-4-nitroanilide. Enzyme activities are expressed in nmol of 5-amino-2-nitrobenzoate per min per g of tissue.

### Glutathione content in aorta

The assay was based on the use of 2,3-naphthalenedicarboxyaldehyde, a glutathione- fluorogenic probe, as previously described (Maguin Gaté et al., 2011). Briefly, thoracic aortic tissue was homogenized in cold 10% (v/v) perchloric acid containing 5 mM EDTA. After centrifugation (14,000 × g for 5 min at 4°C), the acidic supernatants were neutralized with 10 M of NaOH and samples were transferred to a 96-well microtiter plate (Nunc, black model B23806). Then, 0.4 M borate buffer (pH 9.2) and 5.4 mM 2,3-naphthalenedicarboxyaldehyde solution were added into each well and the fluorescence intensity of glutathione-2,3-naphthalenedicarboxyaldehyde adduct was measured within 15 min by using a microplate reader (λ_exc_ = 485 ± 20 nm, λ_em_ = 528 ± 20 nm, Synergy 2 mode, Biotek Instruments, Colmar, France). The glutathione content in samples was calculated from the calibration curve equation, and expressed as nmol glutathione per g of wet tissue.

### Data analysis and statistical tests

Relaxant responses of GSNO, sodium nitroprusside or carbachol were given as the percentage of 10^−6^ M phenylephrine precontraction and calculated as:

$$\%\;relaxation=\left[\frac{\begin{array}{l} (Tension\left(PHE\;10^{-6}\text{M},\;\text{ g}\right)-Tension\\ \;\left(GSNO,\;nitroprusside\;or\;carbachol,\;g\right)) \end{array}}{\left(Tension\;\left(PHE\;10^{-6},g\right)-Tension\;\;\left(BASELINE,\;g\right)\right)}\right]\;\times \;100$$

The half maximal effective concentration (EC_50_) and maximal response (E_max_) were calculated by fitting each individual concentration response curve using the Hill logistic equation (Graph Pad prism® software version 5.0):

$$\%\;relaxation=E_{min}+\frac{\left(E_{max}-E_{min}\right)}{\left(1+10^{\left(\left(logEC_{50}\;-\;concentration\right)\;\times \;Hill\;slope\right)}\right)}$$

where E_min_ and E_max_ = minimal and maximal response reached in each concentration-response curve.

The pD_2_ was calculated as −log EC_50_.

After modeling individual concentration response curve, means ± S.E.M. of E_max_ and pD_2_ were analyzed by a Student *t*-test (adult SHR compared to WKY; SHR-S to WKY-S). We also performed two-ways (hypertension, concentration) ANOVA analysis followed by a *post-hoc* Bonferroni test to compare individual concentrations. The null hypothesis was rejected at *p* < 0.05.

## Results

**Figure 1:**
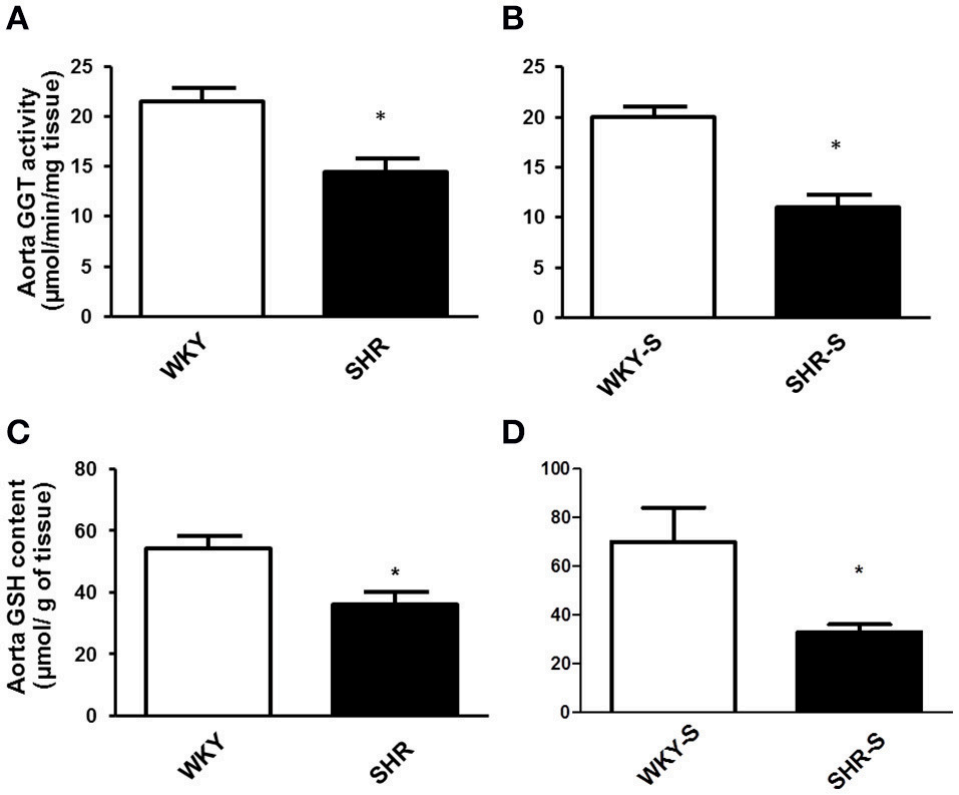
**Influence of hypertension and/or salt diet on aortic GGT activity and aortic glutathione content**. Thoracic aortic GGT activity **(A,B)** and aortic glutathione content **(C,D)** in WKY/SHR rats submitted (SHR-S/WKY-S; **B,D**) or not **(A,C)** to high-salt diet 1% (w/v) during 8 weeks. ^*^p < 0.05 Student *t*-test vs. WKY of the same series. *n* = 3–4 aortic rings per group, isolated from 4 different rats per group.

Thoracic aortic activity of GGT and glutathione content of the aortic wall decreased both by 33% in SHR and by 45 and 53% in SHR-S (Figure 1).

**Figure 2:**
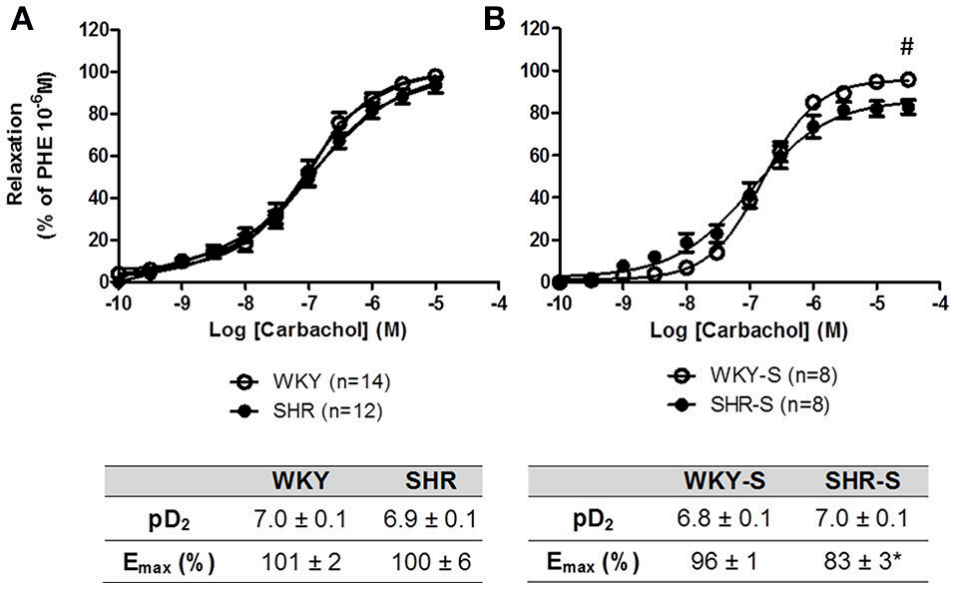
**Influence of hypertension and/or salt diet on the vasorelaxant responses to carbachol**. Cumulative concentration response curves to carbachol of phenylephrine pre-constricted aortic rings isolated from: **(A)** adult SHR/WKY rats and **(B)** adult SHR/WKY rats submitted to high-salt diet 1% (w/v) during 8 weeks (SHR-S/WKY-S). ^*^p < 0.05 Student *t*-test vs. WKY or WKY-S of the same series following the Hill analysis. ^#^*p* < 0.05 vs. WKY or WKY-S at the same concentration, two-way analysis of variance (ANOVA) + Bonferroni *post-test*. *n* = number of aortic rings per group, isolated from 4 to 8 different rats per group.

Each individual concentration response curve to sodium nitroprusside, carbachol and GSNO fitted the Hill model. Responses (E_max_ and pD_2_) to sodium nitroprusside were similar in all groups (Table 1). Neither E_max_ nor pD_2_ for carbachol changed in SHR compared to WKY. E_max_ for carbachol decreased by 14% in high salt diet SHR (*p* < 0.05 vs. WKY-S) with no change in pD_2_ (Figure 2).

**Table 1:** **Influence of hypertension and/or salt diet on the vasorelaxant responses to sodium nitroprussiate**.

		**WKY**	**SHR**	**WKY-S**	**SHR-S**
Sodium nitroprussiate	pD_2_	7.8 ± 0.2	8.3 ± 0.4	8.2 ± 0.2	8.4 ± 0.2
	E_max_,%	109 ± 3	111 ± 4	108 ± 5	112 ± 3

*E_max_ and pD_2_ values obtained from cumulative concentration response curves with sodium nitroprusside in phenylephrine pre-constricted aortic rings isolated from WKY et SHR submitted or not to high-salt diet 1% (w/v) during 8 weeks (SHR/WKY and SHR-S/WKY-S). n = 4–7 aortic rings per group, isolated from 3 different rats per group*.

**Figure 3:**
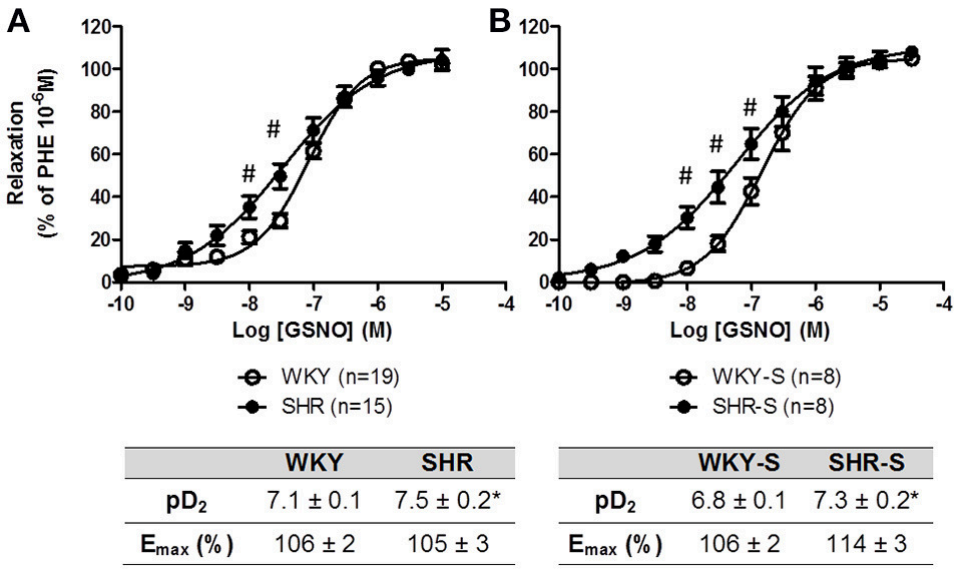
**Influence of hypertension and/or salt diet on the vasorelaxant responses to GSNO**. Cumulative concentration response curves to GSNO of phenylephrine pre-constricted aortic rings isolated from: **(A)** adult SHR/WKY rats and **(B)** adult SHR/WKY rats submitted to high-salt diet 1% (w/v) during 8 weeks (SHR-S/WKY-S). ^*^p < 0.05 Student *t*-test vs. WKY or WKY-S of the same series following the Hill analysis. ^#^*p* < 0.05 vs. WKY or WKY-S at the same concentration, two-way analysis of variance (ANOVA) + Bonferroni *post-test*. *n* = number of aortic rings per group, isolated from 6 different rats per group.

**Figure 4:**
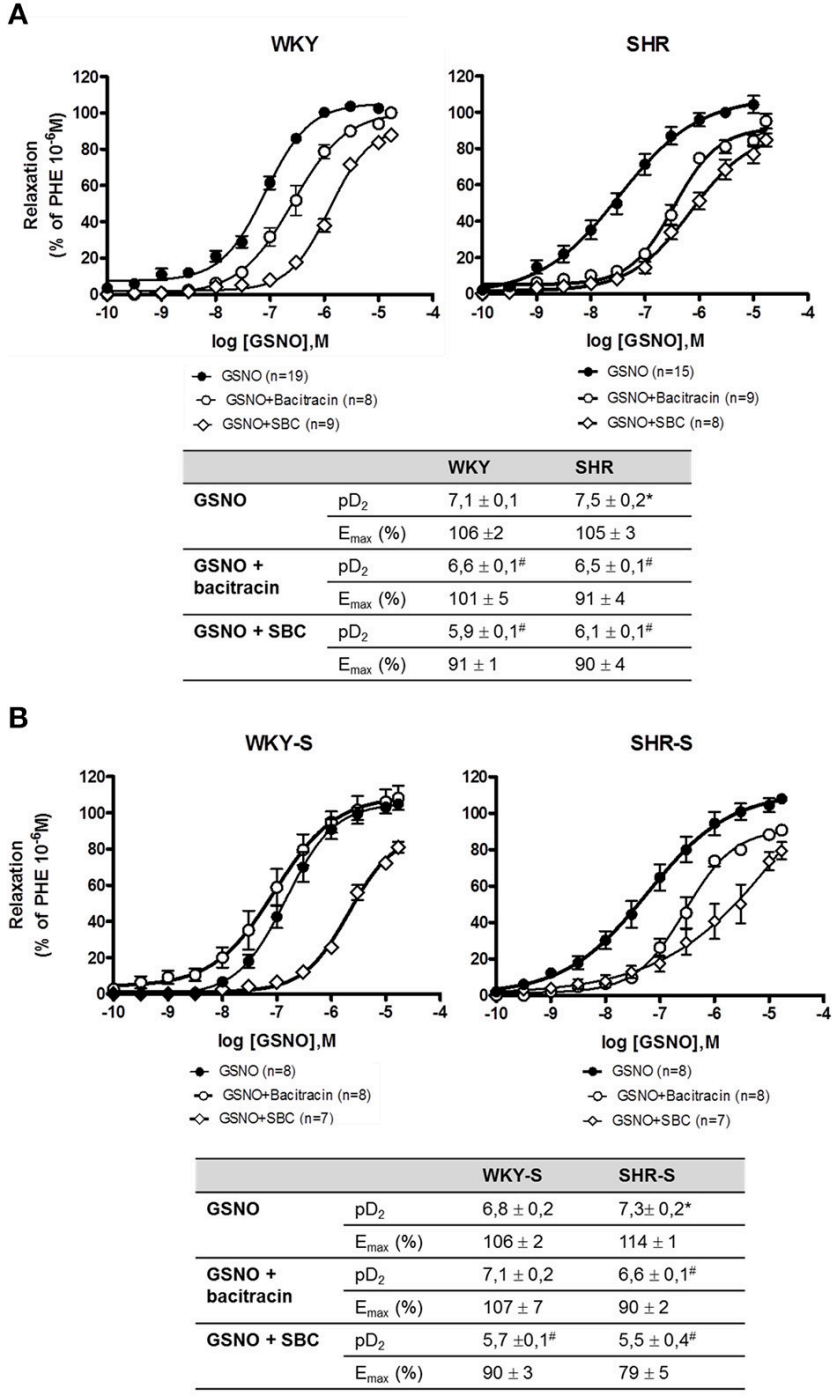
**Impact of serine-borate complex and bacitracin on GSNO-induced vasorelaxations**. Cumulative concentration response curves to GSNO in the presence of serine-borate complex (20 mM) or bacitracin (200 μM) in phenylephrine pre-constricted aortic rings isolated from **(A)** SHR/WKY rats and **(B)** SHR/WKY rats submitted to high-salt diet 1% (w/v) during 8 weeks (SHR-S/WKY-S). ^*^p < 0.05 Student *t*-test vs. WKY or WKY-S of the same series following the Hill analysis. ^#^*p* < 0.05 vs. GSNO alone of the same group, Student *t*-test, *n* = number of aortic rings per group, isolated from 4 to 11 different rats per group.

Concentration response curves to GSNO did not show any decrease in E_max_ with hypertension alone nor with high salt diet as compared to their corresponding control WKY. The concentration response curves were even slightly shifted to lower concentrations of GSNO in SHR (pD_2_ + 0.4) and SHR-S (pD_2_ + 0.5) vs. WKY and WKY-S, respectively (Figure 3). In the presence of serine borate complex (an inhibitor of GGT), or in the presence of bacitracin (an inhibitor of PDI), the concentration response curves to GSNO were shifted to the right in all groups as shown by the significant decreases in pD_2_ (−0.5 to −1.5 log) (Figure 4).

## Discussion

In SHR and SHR-S of the present study, muscle cells dilation capacities (responses to sodium nitroprusside) were maintained, while endothelial NO-dependent vasodilation started to decrease in SHR-S. Aortic GGT activity decreased in SHR and SHR-S, but this did not impair the capacity of GSNO to relax isolated aortic rings. The pD_2_ values for GSNO even increased.

To our knowledge, this is one of the few study, with that of Yilmaz et al., to address the possibility that impairment of NO homeostasis might be related to alterations in plasma (Yilmaz et al., 2013) or in vessel wall GGT activity.

The degree of severity of endothelium dependent NO release vasodilation is commonly evaluated by a decrease in the response to endothelial muscarinic receptor stimulated by carbachol or acetylcholine, in the absence of any modification of the endothelial independent NO vasodilation (no change in response to nitroprusside, as observed in the present study). The extremely abundant literature using such experimental approaches interprets endothelial NO-dependent dysfunction on the basis of a decrease in either pD_2_ or E_max_ or both (Duarte et al., 2001; Demougeot et al., 2005; Bagnost et al., 2008; Kane et al., 2010; Isabelle et al., 2012). In the present study, as those of Isabelle et al. (2012) or Demougeot et al. (2005), 20-week-old adult SHR did not show any decrease in maximal effect for carbachol nor acetylcholine. In salt-treated SHR of the present study, endothelium dependent NO release vasodilation was weak with a −14% decrease in E_*max*_ for carbachol. It has to be noticed that global endothelial dysfunction in SHR not only involves decreased NO bioavailability but also decreased production of endothelium-hyperpolarizing factor and prostacyclin (Deanfield et al., 2005; Schini-Kerth et al., 2011), higher production of endothelium-dependent vasoconstrictive agents (Touyz and Schiffrin, 2004; Félétou et al., 2009) and possible long term structural changes (Touyz and Schiffrin, 2004; Lee and Griendling, 2008). For other cardiovascular diseases, such as atherosclerosis, the prominent feature for endothelial dysfunction lies on the reduction in NO bioavailability, which is regarded as a major factor in the pathogenesis of the disease (Mudau et al., 2012; Jensen and Mehta, 2016; Vanhoutte et al., 2016).

Several recent studies have implicated the GGT activity in cardiovascular diseases. In particular, increased levels of GGT in serum have been correlated with hypertension (Mason et al., 2010) as well as with impaired aortic elasticity in prehypertensive patients (Celik et al., 2010). As GGT is implicated in GSNO catabolism and NO release (Hogg et al., 1997; Bramanti et al., 2009), it is possible that increasing levels of serum GGT may reduce the bioavailability of GSNO in blood, thus hampering its peripheral utilization for regulation of vascular tone. We have previously shown that increasing levels of GGT activity in blood do correspond to increased rates of clearance of exogenously added GSNO (Bramanti et al., 2009). Another study conducted in patients with chronic kidney disease showed increased serum GGT activity in association with endothelial dysfunction (Yilmaz et al., 2013).

Besides the soluble enzyme found in serum, GGT is also present at cellular level in vascular tissues. Substantial levels of the enzyme are expressed in arterial endothelium (Cotgreave and Schuppe-Koistinen, 1994). We have previously shown that such GGT activity is involved in the utilization of GSNO, in that it promotes the local release of NO from GSNO thus mediating its vasorelaxant effect (Dahboul et al., 2012). In atherosclerotic lesions, GGT was found to accumulate in plaques, in association with cells of the macrophagic lineage as well as with the lipid core (Paolicchi et al., 2004; Franzini et al., 2009). This accumulation may derive both from insudation of circulating GGT and from cellular macrophages dependent release of GGT. Moreover, several GGT fractions may be involved (Franzini et al., 2008). Finally, the relationship between tissue and circulating GGT are complex, especially in a pathological environment associating inflammation (atherosclerosis) and/or oxidative stress (present).

A loss of GGT activity was described in endothelial cells submitted to oxidative stress (Muruganandam et al., 2011). Therefore, the pro-oxidant environment associated with hypertension, as witnessed by the 33–53% fall in aortic wall glutathione content in SHR and SHR-S, could participate in the decrease in aortic wall GGT activity we observed. However, this decrease in aortic wall GGT activity did not impair the vasorelaxant effect of GSNO. Furthermore, GSNO-mediated vasodilation was still dependent on GGT, as shown by our experiments under inhibition with serine-borate complex. This suggests that (i) even if it decreases, the GGT activity remains sufficient to maintain GSNO vasorelaxant effect and/or (ii) other pathway of GSNO bioactivation are substituting for GGT. Membrane PDI, a membrane enzyme from the redoxins family, which is involved in the release of NO from GSNO, and in GSNO vasorelaxant effect (Heikal et al., 2011), increases its expression under oxidative stress (Janiszewski et al., 2005; Gaucher et al., 2013). SHR of the present study developed oxidative stress (decreased GSH content, Figure 1) (e.g., through upregulation of NOX; Tabet et al., 2008; Montezano and Touyz, 2012). Moreover, inhibition of PDI with bacitracin decreased the pD_2_ values of GSNO in SHR and SHR-S demonstrating that the vasorelaxant effect of GSNO was dependent on PDI. Therefore, the decreased GGT activity occurring during hypertension may be balanced by an oxidative stress-related increase in PDI expression and/or activity, slightly improving vasorelaxant effects of GSNO in SHR and SHR-S.

One limitation of this study relies on the use of bacitracin. Bacitracin is not a specific inhibitor of PDI, which displays several activities (oxidoreductase, chaperone, and isomerase, Khan and Mutus, 2014). However, most of those activities are involved in the release of NO from GSNO (Shah et al., 2007).

## Conclusion

In conclusion, our data suggest that hypertension is accompanied by a decreased activity of aortic GGT, one of the enzymes catalyzing the release of bioactive NO from GSNO. Nevertheless, vasorelaxation induced by GSNO is slightly improved. We suggest that under hypertension and oxidative stress, GSNO effect is likely warranted by the concomitant action of PDI, the other major (oxidative stress-inducible) enzyme involved in NO release from GSNO.

## Author contributions

MP and FD performed the experiments, acquired data and interpreted the results. CP designed the work, acquired data, interpreted the results and drafted the manuscript. IL designed the work, interpreted the data, drafted, and revised the manuscript. AP and PL designed the work and revised the manuscript. All authors gave final approval of the manuscript to be published.

## Funding

This work was supported by the French Ministry of Education, Research and Technology (Paris, France, EA3452), Région Lorraine and Université de Lorraine (France, project AO186 “Rôles des enzymes impliquées dans la libération de NO à partir des *S*-nitrosothiols au cours de l'hypertension artérielle”).

### Conflict of interest statement

The authors declare that the research was conducted in the absence of any commercial or financial relationships that could be construed as a potential conflict of interest.

## Abbreviations

EDTA: Ethylenediaminetetraacetic acid
EC_50_: half maximal effective concentration
E_max_: maximal response
GGT: gamma-glutamyltransferase
GSH: glutathione
GSNO: *S*-nitrosoglutathione
NO: nitric oxide
PDI: protein disulfide isomerase
WKY: Wistar Kyoto rat
WKY-S: high salt diet submitted Wistar Kyoto rat
SEM: *standard* error of mean
SHR: spontaneous hypertensive rat
SHR-S: high salt diet submitted spontaneous hypertensive rat.
